# Liquid biopsies to monitor and direct cancer treatment in colorectal cancer

**DOI:** 10.1038/s41416-022-01769-8

**Published:** 2022-03-09

**Authors:** Gianluca Mauri, Pietro Paolo Vitiello, Alberto Sogari, Giovanni Crisafulli, Andrea Sartore-Bianchi, Silvia Marsoni, Salvatore Siena, Alberto Bardelli

**Affiliations:** 1 IFOM-FIRC Institute of Molecular Oncology, Milan, Italy; 2 Department of Oncology and Hemato-Oncology, Università degli Studi di Milano, Milan, Italy; 3 Candiolo Cancer Institute, FPO–IRCCS, 10060 Candiolo, TO Italy; 4 Department of Oncology, University of Torino, 10060 Candiolo, TO Italy; 5 Department of Hematology, Oncology, and Molecular Medicine, Grande Ospedale Metropolitano Niguarda, 20162 Milan, Italy

**Keywords:** Tumour biomarkers, Cancer genetics

## Abstract

Colorectal cancer (CRC) is one of the most prevalent and deadly cancers worldwide. Despite recent improvements in treatment and prevention, most of the current therapeutic options are weighted by side effects impacting patients’ quality of life. Better patient selection towards systemic treatments represents an unmet clinical need. The recent multidisciplinary and molecular advancements in the treatment of CRC patients demand the identification of efficient biomarkers allowing to personalise patient care. Currently, core tumour biopsy specimens represent the gold-standard biological tissue to identify such biomarkers. However, technical feasibility, tumour heterogeneity and cancer evolution are major limitations of this single-snapshot approach. Genotyping circulating tumour DNA (ctDNA) has been addressed as potentially overcoming such limitations. Indeed, ctDNA has been retrospectively demonstrated capable of identifying minimal residual disease post-surgery and post-adjuvant treatment, as well as spotting druggable molecular alterations for tailoring treatments in metastatic disease. In this review, we summarise the available evidence on ctDNA applicability in CRC. Then, we review ongoing clinical trials assessing how liquid biopsy can be used interventionally to guide therapeutic choice in localised, locally advanced and metastatic CRC. Finally, we discuss how its widespread could transform CRC patients’ management, dissecting its limitations while suggesting improvement strategies.

## Introduction

Colorectal cancer (CRC) is the third most common and the second most deadly cancer worldwide, representing 10.2% of new cases and 9.2% of cancer-related deaths [1]. Overall survival (OS) at 5-year from initial diagnosis spans from 87–90% in stage I–II to 68–72% in stage III, lowering to 11–14% in stage IV metastatic CRC (mCRC) [2]. Today, treatment algorithms in CRC are mainly driven by cancer staging, patients’ performance status and molecular profiling encompassing *RAS*, *BRAF, ERBB2* and mismatch repair (MMR) status assessed on surgical or core biopsy tumour samples [3, 4]. In addition, following tumour-agnostic drug approvals, new molecular biomarkers such as *NTRK1-3* translocations and high tumour mutational burden (TMB) have emerged in CRC as well as in other malignancies [5, 6]. Differently from the metastatic setting, with the exception of microsatellite instability (MSI), in stages II and III CRC there is still a lack of validated biomarkers identifying those patients more likely to benefit from adjuvant cytotoxic regimens [7]. This applies also following resections of metastatic disease, where usually post-operative treatments are administered despite the lack of prognostic biomarkers [3, 4]. To date, all molecular analyses exploited for clinical decision-making are based on core tumour biopsies, which currently represent the gold standard as per clinical guidelines [3, 4]. However, single solid tissue snapshots have several limitations such as tumour spatial and temporal heterogeneity, and technical feasibility issues [8–12]. In this clinical scenario, liquid biopsy (LB) is increasingly gaining attention as a complementary and potentially alternative non-invasive tool to bypass these limitations [13–16].

The term “liquid biopsy” defines the collection of tumour-derived biomarkers in the blood or other body fluids, such as urine, saliva, stool or cerebrospinal fluid [17–19]. Circulating tumour DNA (ctDNA), circulating tumour cells (CTCs) and exosomes are the most common tumour-related biomarkers assessed on liquid biopsy so far [8, 17]. Among these, ctDNA analysis, consisting in the isolation of DNA fragments from the bloodstream of patients, has already shown its potential in capturing the CRC molecular complexity, coupled with the technical advantages of minimal invasiveness and fast turnaround time [17, 20]. Assessment of real-time tumour-associated genomic changes and treatment selection can be guided by ctDNA monitoring in non-metastatic as well as metastatic CRC. Particularly, in mCRC the omni-comprehensiveness of ctDNA could replace solid tumour tissue biopsy as a more accurate tool in high burden diseases, potentially refining treatment tailoring such as rechallenge with anti-EGFR drugs [12, 17, 20, 21]. Given the promising retrospective data in all disease stages, validation of the interventional ctDNA role to drive treatment decisions is presently been tested to translate this tool into everyday clinical practice.

In this review, we will first discuss retrospective evidence supporting the role of ctDNA in CRC. Secondly, we will analyse currently ongoing trials testing interventional ctDNA as a tool to drive clinical decision-making in CRC focusing on initially available prospective data in this field. Finally, we will provide potential solutions to overcome current limitations for the incorporation of liquid biopsy in the management of CRC.

## Circulating tumour DNA in colorectal cancer

CRC is one of the solid tumours shedding the highest amount of ctDNA in the bloodstream [22, 23]. However, since the ratio between ctDNA and circulating-free DNA (cfDNA) can greatly vary between less than 1% and more than 40%, due to many variables, including the location of the primary tumour and metastases, ctDNA detection requires highly sensitive and specific approaches [8, 12, 24]. Available assays for the detection of ctDNA have steadily increased in sensitivity and specificity. Indeed, today high-resolution PCR-based technologies, such as BEAMing, reached a sensitivity up to 0.001% with a specificity up to a single base difference, establishing the limit of detection (LOD) as 1 copy of mutant DNA/mL. It is also necessary to consider that the LOD depends on the chromosomal region (polymorphism adjacent to the hotspot could decrease the LOD) and the specificity of the probes [25, 26]. PCR-based tests, however, can detect only a few loci, while next-generation sequencing (NGS) can capture the full spectrum of genetic alterations, including Single Nucleotide Variants (SNVs), copy number variations and chromosomal rearrangements, depending on sequencing library and bioinformatic tools exploited [27]. Whole-genome sequencing (WGS) can further broaden the genomic analysis but with higher costs and lower sensitivity, given the difficulty in systematically applying barcoding systems required to increase sensitivity, especially for low-frequency ctDNA [28, 29]. In addition, if a low LOD cannot be achieved by WGS, Cristiano and colleagues developed an approach that combines the high fragmentation patterns of cell-free DNA in the genome of cancer patients with the mutation-based cell-free DNA analyses to detect 91% of patients with cancer from a healthy donor, obtaining 81% sensitivity and 95% specificity in CRC [30]. This integrated approach stood as a proof-of-concept that WGS from cfDNA could be exploited for screening, early detection and monitoring of human cancer [30]. Broadening the spectrum of genetic alterations assessable on ctDNA, methylation-specific PCR and methyl-BEAMing were found capable of detecting methylated ctDNA. However, a deeper discussion concerning the method of ctDNA detection, which is beyond the objectives of this review, is already discussed in other published reviews [8, 13, 20].

**Fig. 1:**
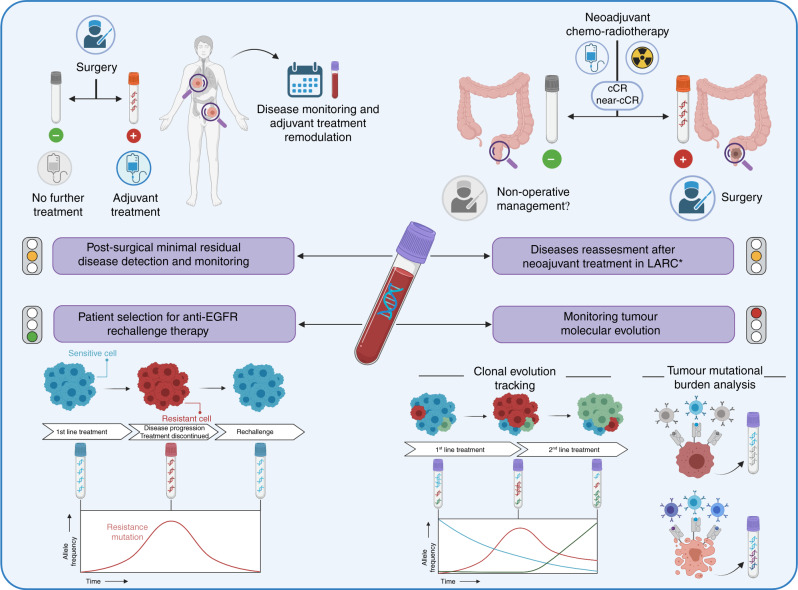
Interventional liquid biopsy can orient therapeutic decision-making in CRC. Circulating tumor DNA (ctDNA) analysis through liquid biopsy has proven to be a robust method to tailor personalised treatments for CRC) patient care. Promising results have been achieved in the post-surgical adjuvant setting and in driving treatment choice in locally advanced rectal cancer (LARC) after neoadjuvant treatment. Ongoing and future studies, exploiting ctDNA to guide anti-EGFR rechallenge therapy and treatment choices based on mutational and molecular CRC evaluation, will further expand the use of interventional liquid biopsy in CRC patients care. The “traffic lights”, close to each box where clinical strategies are defined, summarise the level of evidence supporting applicability of interventional ctDNA in the clinical practice. Figure created with BioRender.com. *Keys*: LARC locally advanced rectal cancer, * ctDNA negativity or positivity might be taken into account in patients treated with neoadjuvant multimodal treatment and achieving near clinical complete response (cCR) or cCR to evaluate if they might be candidate to non-operative management rather than curative surgery, respectively; cCR clinical complete response, EGFR epidermal growth factor receptor, green light = initial prospective data available, orange line = only retrospective data available, red light = only partial data available.

Given its potential clinical utility and improvements in technical applicability, ctDNA isolation through liquid biopsy has actively been pursued as a potential tool to refine CRC patients care (Fig. 1). Following, we discuss the clinical settings where the use of liquid biopsy can support clinical decision-making and guide therapeutic choices.

## Colorectal cancer screening and diagnosis

The idea of exploiting cfDNA derived from cancer cells as a non-invasive screening technique is as old as the discovery of ctDNA itself [31]. It stems from the observation that CRC patients display higher levels of mutated DNA in their bloodstream compared to healthy controls [32]. The major limitation to its widespread clinical use is the suboptimal limit of detection for small invasive cancers or precancerous lesions, which characterise a relevant target of the screening campaigns [33, 34]. Moreover, since no information about the mutational profile of the cancer is available in the screening setting, panels of frequently mutated genes in CRC need to be verified to achieve an acceptable sensitivity [35], even if this approach also increases the risk of false-positive results due, for instance, to clonal haematopoiesis [36, 37]. A potential option to overcome this limitation is to associate already-known cancer protein biomarkers to mutational panels, as investigated in the CancerSEEK study, that integrated both mutations in a panel of 16 genes (in 1933 genomic positions) and 8 protein biomarkers from plasma, with a good performance in identifying 8 common cancer types [38]. Interestingly, even if the sensitivity for CRC is only around 60%, the specificity of the test is more than 99%, rendering this test a good candidate for multi-cancer screening before more invasive diagnostic procedures [38, 39].

In order to bypass limitations due to false-positive results, many approaches in this setting have focused on specific features of cancer genomes to be used as biomarkers [40, 41]. The first approach involves the study of the methylation pattern of cancer genomes compared to DNA derived from healthy cells [40, 42]. In particular, this approach may involve the evaluation of a single gene, such as the FDA-approved EpiproColon test which uses Septin9 (*SEPT9*) gene methylation with a sensitivity and a specificity of 68–72% and 80–82%, respectively [43–45], or the ColoSure test which evaluates vimentin (*VIM*) methylation state with comparable diagnostic accuracy [46]. On the other hand, the evaluation of thousands of methylation sequences using targeted bisulfite sequencing with the integration of machine learning to predict cancer-associated patterns of methylation is the approach exploited by GRAIL that, in the ambitious STRIVE study (NCT03085888), aims at identifying ctDNA from 12 tumour types including CRC with remarkable sensitivity and specificity (60–73% and >98%, respectively) in the first retrospective case-control study [47]. Another approach for the detection of cancer-specific circulating DNA relies on the analysis of the DNA fragments’ length (i.e. fragmentomics), which has proven valuable in identifying seven common cancer types including CRC [30], and is currently exploited in the DELFI study (NCT04825834). The lessons deriving from the study of ctDNA methylation and fragmentation have converged in the design of multimodality ctDNA test LUNAR-2, which has recently shown high values of sensitivity and specificity, also for stage I–II disease, of 88% and 94%, respectively [48, 49].

Finally, analysis of cfDNA derived from stool has been proposed as another means to increase the sensitivity of non-invasive faecal immunohistochemical test (FIT), particularly in early tumour stages and in precancerous lesions in which classical faecal tests display poor sensitivity [50–52]. Specifically, in a randomised clinical trial of multitarget stool DNA testing the sensitivity of detecting CRC versus FIT was improved from 74 to 92%, and the rate of detection of polyps with high-grade dysplasia was also dramatically increased from 46 to 69%, though the rate of false-positive was higher for the stool DNA test versus FIT [51]. Collectively, although the use of cfDNA for CRC screening is valuable and increasingly cheaper, it is still too early to claim its prime time in the clinic. Thus, based on available data, cfDNA analysis could only be exploited on top of clinically validated screening tools.

## Post-surgical resection with curative intent

Surgery represents the main curative treatment of CRC patients with localised, locally advanced and also oligometastatic disease [3, 4]. In these patients the presence of ctDNA in the blood post-surgery can identify the existence of a minimal residual disease (MRD), invisible at radio-imaging and conceptually similar to the MRD in hematology [37, 53].

### Post-surgical liquid biopsy in localised and locally advanced stages

In pivotal retrospective studies, a positive post-surgical liquid biopsy at a short distance after curative-intent surgery forebodes a disease recurrence within 2 years in almost all cases (Table 1 and Fig. 1). More than a decade ago, Diehl and co-workers first demonstrated that, in stage II–IV CRC patients who had undergone surgery with curative intent, median ctDNA decreased by 97% in less than a day and by 99% within 10 days. On the opposite, if supposedly curative resection was not attained, ctDNA levels decreased much less evidently or increased [54]. After this first evidence, other studies confirmed that the ctDNA was capable of predicting recurrence in CRC patients after surgical resections for localised (stage I–III) or oligometastatic disease (Table 1) [21, 55–60].

**Table 1:** Main published retrospective and interventional studies suggesting the potential clinical role of circulating tumour DNA (ctDNA) assessment in colorectal cancer patients.

Study	Disease stage	ctDNA method	N.° Pts.	Colon/rectal	Main findings
(A) *Post-surgical resection with curative intent*					
Tie et al. 2016 [57]	II	Safe-SeqS assay	231	231/0	• Pts after surgery not receiving adjuvant chemotherapy: post-operative ctDNA detection correlates with higher risk of recurrence (HR 18; 95% CI 7.9–40) • Pts receiving adjuvant cytotoxic regimens: ctDNA positivity after treatment correlates with an inferior RFS (HR 11; 95% CI 1.8–68).
Diehl et al. 2008 [54]	II–IV	Real-time PCR	20	NA	• Worse RFS in ctDNA-positive pts after surgery (*p* = 0.006).
Tie et al. 2021 [59]	IV (CRLM resections)	Safe-SeqS) assay	54	NA	• ctDNA post-operative positive pts had lower RFS (HR 6.3; 95% CI 2.58–15.2) and OS (HR 4.2; 95% CI 1.5–11.8). • ctDNA clearance observed in 3 pts receiving post-operative treatment, 2 of whom remained disease-free. • End-of-treatment (surgery ± adjuvant cytotoxic regimen) ctDNA positivity was associated with 0% 5-year RFS compared to 75.6% in those ctDNA-negative (HR 14.9; 95% CI 4.94–44.7).
Tie et al. 2019 (a) [58]	III	Safe-SeqS) assay	96	96/0	• Positive ctDNA post-surgery correlates with an inferior RFS (HR 3.8; 95% CI, 2.4–21.0). • Positive ctDNA post-adjuvant therapy: lower 3-year RFI (HR, 6.8; 95% CI, 11.0–157.0). • Post-surgical ctDNA status is independently associated with RFI after adjusting for clinicopathologic risk factors (HR, 7.5; 95% CI, 3.5–16.1).
Reinert et al. 2016 [55]	I–IV	ddPCR	11	5/6	• 6/6 post-surgery positive ctDNA pts relapsed while 0/5 ctDNA-negative did (8 pts were ctDNA-positive prior to surgery but 1 was stage 1 CRC pts and 2 were stage 2 CRC pts).
Reinert et al. 2019 [56]	I–III	HiSeq 2500 system, Illumina Inc	125	119/6	• Pre-operative ctDNA-positive pts were 108/122 (88.5%). • Post-operative ctDNA-positive pts were more likely to relapse (HR 7.2; 95% CI, 2.7–19.0). • Positive ctDNA pts after adjuvant cytotoxic regimens were more likely to relapse (HR 17.5; 95% CI, 5.4–56.5). • 3/10 ctDNA-positive pts were cleared by adjuvant regimens • In all multivariate analyses, ctDNA positivity was independently associated with relapse after adjusting for known clinicopathologic risk factors.
Parikh et al. 2021 [21]	I–IV	Guardant Reveal, Health panel	84	54/30	• PPV in determining disease recurrence in ctDNA-positive pts: 100% • Integrating epigenomic signatures increased sensitivity by 25–36% versus genomic alterations alone.
Taieb et al. 2019 [60]	II–III	ddPCR	805	NA	• 2-year DFS: 64% in ctDNA-positive versus 82% in those negative (HR 1.75; 95%CI 1.25–2.45). • In the multivariate analysis including age, gender, MSI, perforation, T stage, N stage and treatment arm (3 vs 6 months adjuvant treatment) ctDNA was confirmed an independent prognostic marker (HR 1.85 95% CI 1.31–2.61).
Kotaka et al. 2022 [62]	I–IV	Signatera bespoke multiplex-PCR NGS assay	1564	NA	• Post-operative ctDNA positivity at 4 weeks after surgery was associated with an inferior DFS (HR 10.9; 95% CI 7.8–15.4). • DFS rates by ctDNA dynamics at 4 and 12 weeks postoperatively were significantly different between “positive to negative” versus “positive to positive” (HR 15.8; 95% CI 5.7–44.2). • Adjuvant treatment improved DFS in stages II, III and IV (analysed separately) among ctDNA-positive patients, while those ctDNA-negative do no benefit from medical post-operative treatment (HR 1.3; 95% CI 0.5–3.6).
(B) *Neoadjuvant setting*					
Tie et al. 2019 (b) [74]	II–III	Safe-SeqS) assay	159	0/159	• HR for recurrence in ctDNA-negative vs ctDNA-positive after pre-operative chemotherapy: HR6.6 (*p* < 0.001); HR for recurrence in ctDNA-negative vs ctDNA-positive after surgery: HR 13.0 (*p* < 0.001). • 3-year RFS rate: 33% for the post-operative ctDNA-positive patients versus 87% for the post-operative ctDNA-negative patients, irrespective of clinicopathological risk factors (HR 6.0; *p* < 0.001).
Vidal et al. 2021 [75]	II–III	NGS (Guardant Reveal)	72	0/72	• Detectable pre-surgery ctDNA after chemotherapy significantly associated with systemic recurrence, shorter DFS (HR 4; *p* = 0.033), and shorter OS (HR 23; *p* < 0.0001). • No significant association between ctDNA status and pathologic response.
McDuff et al. 2021 [76]	II–III	NGS (Guardant Reveal)	29	0/29	• Overall margin-negative, node-negative resection rate: 88% in undetectable pre-operative ctDNA versus 44% in patients with detectable pre-operative ctDNA (*p* = .028). • Relapse for post-operative ctDNA-positive versus ctDNA-negative: 100% vs 13.3% (HR = 11.56; *p* = .007).
Khakoo et al. 2020 [77]	II–III	ddPCR	47	0/47	• ctDNA status after CRT Associated with primary tumour response by mrTRG (*p* = 0.03). • MFS significantly shorter in ctDNA-positive patients after completing CRT (HR 7.1; *p* < 0.001), ctDNA-positive pre and mid-CRT (HR 3.8; *p* = 0.02), and ctDNA-positive pre, mid, and after CRT (HR 11.5; *p* < 0.001) versus ctDNA-negative or non-persistent.
Zhou et al. 2020 [80]	II–III	NGS (Targeted capture sequencing)	106	0/106	• Pre-operative ctDNA-positive rate significantly lower in: patients with better pathologic tumour regression grade (ypCAP 0–1 vs ypCAP 2–3; *p* < 0.001), patients with pCR vs non-pCR (*p* = 0.02), patients with lower pT stage (ypT 0–2 vs ypT 3–4; *p* = 0.002). • ctDNA positivity at every timepoint after the start of neoadjuvant treatment is associated to shorter MFS (*p* < 0.05).
Wang et al. 2021 [79]	II–III	NGS (Targeted capture sequencing)	119	0/119	• ctDNA clearance after CRT associated with a low probability of non-pCR (OR = 0.11, *p* = 0.04). • Incorporation of ctDNA and mrTRG after CRT exhibits high performance in predicting pCR (AUC = 0.886). • ctDNA-positive pts display a worse RFS after surgery (HR = 9.29; *p* < 0.001).
Murahashi et al. 2020 [78]	II–III	Amplicon-based deep sequencing	85	0/85	• Change in ctDNA predicts pCR after pre-operative therapy (*p* = 0.0276). • Post-operative ctDNA detection predicts recurrence (*p* = 0.0127).
(C) *Anti-EGFR rechallenge in the metastatic setting*					
Cremolini et al. 2018 [90]	IV	ddPCR and Ion Torrent S5 XL	28	22/6	• No *RAS* mutations on ctDNA in patients achieving PR • *RAS* wt on ctDNA patients had longer PFS than those *RAS* mutated on ctDNA (median PFS 4.0 vs 1.9 months; HR, 0.44; 95% CI, 0.18–0.98; *p* = .03).
Martinelli et al. 2021 [91]	IV	Idylla qPCR	77	52/25	• *RAS/BRAF* wt on ctDNA patients had 17.3 months mOS (95% CI, 12.5–22.0 months) compared with 10.4 months (95% CI, 7.2–13.6 months) in those with mutated ctDNA (HR 0.49; 95% CI, 0.27–0.90; *p* = .02). • mPFS was 4.1 months (95% CI, 2.9–5.2 months) in *RAS/BRAF* wt patients compared with 3.0 months (95% CI, 2.6–3.5 months) in patients with mutated ctDNA (HR, 0.42; 95% CI, 0.23–0.75; *p* = .004)
Sartore-Bianchi et al. 2021 [92]	IV	ddPCR	27	22/5	• Among pts with *RAS*, *BRAF* and *EGFR* ECD wt mCRC prospectively interventionally assessed by ctDNA achieved RR 30%, DCR 63% and mPFS 16.4 weeks with panitumumab rechallenge monotherapy.

*N.°* number; *pts*. patients, *RFS* relpase-free survival, *OS* overall survival, *HR* hazard ratio, *ctDNA* circulating tumour DNA, *RFI* relapse-free interval, *CRC* colorectal cancer, *PCR* polymerase chain reaction, *ddPCR* droplet-digital PCR, *PPV* predictive positive value, *DFS* disease free survival, *DFI* disease-free interval, *MSI* microsatellite instability, *T* primary tumour stage according to TNM classification, *N* lymph-nodes status according to TNM classification, *RFS* recurrence-free survival, *NGS* next-generation sequencing, *CRT* chemoradiation therapy, *pCR* pathological complete response, *ypCAP* post chemoradiation outcome according to the College of American Pathologists system, *MFS* metastasis-free survival, *mrTRG* tumour regression grade assessed by magnetic resonance, *wt* wild-type, *AUC* area under the curve, *PR* partial response, *PFS* progression-free survival, *mOS* median overall survival, *mPFS* median progression-free survival, *ECD* ectodomain, *RR* response rate, *DCR* disease control rate.

The prognostic role of ctDNA is specifically dramatic in high-risk stage II (T4) and stage III CRC patients. So far, due to the lack of truly predictive markers, the standard of care (SoC) for these patients is fluoropyrimidine ± oxaliplatin-based regimens despite the fact that approximately 50% of cases are cured by surgery alone, and chemotherapy is providing a relatively small net survival advantage of around 3–5% and 10–15%, respectively [7, 61]. In the last years the question has been: can ctDNA safely cherry-pick only patients that have a post-surgery MRD and thus should receive adjuvant treatment, while sparing treatment and toxicities to those already rendered disease-free by surgery alone? In untreated stage II CRC patients, ctDNA positivity indeed predicts a higher risk of recurrence with unprecedented double-digit probability statistics (HR 18; 95% CI 7.9–40) [57]. Moreover, in the same clinical context, ctDNA positivity is also associated with worse relapse-free survival (RFS) among those patients who had adjuvant treatment [57]. Later, the same authors demonstrated that ctDNA positivity was remarkably prognostic also in stage III patients, predicting disease relapse both post-surgery and post-adjuvant treatment [58]. A retrospective analysis in 805 adjuvant chemotherapy-treated stage III patients enrolled in the IDEA trial showed a worse 2-year disease-free survival (DFS) in ctDNA-positive patients if compared to negative ones (64% versus 84%, HR 1.75 95% CI 1.25–2.45) [60]. In addition, ctDNA positivity after surgery with curative intent in localised and locally advanced CRC independently correlates with a worse RFS even after adjusting for clinicopathological risk factors [55, 56, 60]. At the recent 2022 ASCO Gastrointestinal Cancer Symposium, the GALAXY study confirmed the ctDNA prognostic role in more than 1500 all stages surgically resected CRC patients [62]. Kotaka and collaborators also demonstrated that DFS rates by ctDNA dynamics 4 to 12 weeks after surgery were significantly different between “positive to negative” versus “positive to positive” (HR 15.8; 95% CI 5.7–44.2) [62]. More interestingly, they found that in ctDNA-positive patients adjuvant treatment improved 6 and 12 months DFS in stages II, III and IV, while not providing any DFS advantage in ctDNA-negative ones (HR 1.3; 95% CI 0.5–3.6) [62]. Finally, ctDNA-guided treatment in the stage II CRC adjuvant setting appears to be a cost-effective strategy aiming at reducing overtreatment in this specific setting [7, 63].

### Post-surgical liquid biopsy in oligometastatic disease

In selected stage IV oligometastatic patients, surgical resection of metastasis can be pursued with curative intent [3, 4, 64]. In this setting as well, ctDNA provided striking results comparable to those described above for non-metastatic disease (Table 1). Diehl and co-workers first reported the prognostic potential of ctDNA detection in 20 patients with the liver-limited disease treated by partial hepatectomy. Of these, 16 were ctDNA-positive after surgery and all but one relapsed while those ctDNA-negative did not [54]. More recently, Tie and co-workers demonstrated that patients with liver-only metastases undergoing surgical resection had a lower RFS and survived less in the case of ctDNA positivity [59]. Of note, ctDNA clearance was achieved in 2 out of 3 patients receiving post-operative treatment supporting the effectiveness of post-operative treatments in this setting [59].

Despite this exciting amount of retrospective evidence suggesting that ctDNA is a potential predictive marker of disease recurrence in radically resected stage I–IV CRC patients, the actual clinical benefit is yet to be proven in prospective interventional trials. We reviewed (using ClinicalTrial.gov) ongoing trials aiming to verify if the assessment of ctDNA on plasma might be exploited interventionally in treating CRC patients. This search was performed in October 2021 and the Medical Subject Headings terms used were (“Colo-rectal Cancer” as condition/disease) and (“circulating tumor dna” as other terms) (Table 2). Particularly, several ctDNA-guided clinical trials are ongoing in the post-surgical setting exploiting different approaches and designs (Table 2). As an example, in the ongoing PEGASUS trial 140 high-risk stage II and stage III MSS patients are being ctDNA screened within a month from surgery and the intensity of their adjuvant treatment is modulated by the results (NCT04259944). ctDNA-negative patients receive capecitabine monotherapy while positive receive CAPOX for 3 months. In the LB-negative arm a series of shortly spaced ctDNA assessments are used to minimise the risk of false-negative, allowing to rapidly escalate to CAPOX whenever this is necessary. In the LB positive arm patients are re-biopsied for ctDNA at the end of the third month of capecitabine plus oxaliplatin (CAPOX regimen) and, if negative, they are de-escalated to a maintenance period with capecitabine, while if still positive they are switched to 5-fluorouracil plus irinotecan (FOLFIRI regimen) that is the SoC combination treatment in the setting of resistance to adjuvant treatment [3, 4]. In addition, the ALTAIR (NCT04457297) trial is investigating the role of trifluridine/tipiracil versus placebo in the case of stage II–IV resected ctDNA-positive CRC patients, while the VEGA (jRCT1031200006) study is assessing if surgery alone is non-inferior to adjuvant capecitabine plus oxaliplatin (CAPOX) in ctDNA-negative patients [65]. Other trials such as the MEDOCC-CrEATE [66], DYNAMIC-II and COBRA are focusing on stage II CRC patients to assess ctDNA role in driving treatment decision-making (Table 2). In stage III CRC instead, the DYNAMIC-III (ACTRN12617001566325) trial is aiming at assessing whether a treatment escalation strategy based on ctDNA might be superior to the standard of care in terms of RFS (Table 2). Overall, several studies are currently ongoing trying to prospectively assess the interventional role of ctDNA in stages I–III CRC through different experimental strategies and designs (Table 2). As depicted in the upper left box of Fig. 1, ctDNA might dramatically impact future post-operative treatment algorithms on top of main clinicopathological variables such as tumour staging and resection margins status. Indeed, radically resected CRC patients with no ctDNA in their plasma might be spared from receiving adjuvant treatments, while those positive for ctDNA will be candidates to receive adjuvant or an even more intensive treatment regimen. Furthermore, when results from these studies will be available, meta-analyses encompassing the range of different studies in this setting will be warranted to derive robust and potentially practice-changing conclusions.

**Table 2:** Main ongoing clinical trials (*N* = 21) investigating the role of interventional circulating tumour DNA (ctDNA) to drive treatment decision-making in colorectal cancer (CRC) patients according to different clinical settings, suggesting potential future applications and developments.

Study (Code Identifiers) Location	Trial design Status	Estimated enrolment (N pts) ctDNA analysis	Main characteristics and inclusion criteria
*Post-surgical resection with curative intent*			
PEGASUS (NCT04259944) Italy–Spain	Phase II^a^ Recruiting	140 LUNAR1 panel	• Resected stage III or T4N0 stage II colon cancer • ctDNA-guided adjuvant treatment: initially those ctDNA-positive will receive CAPOX while those negative capecitabine monotherapy; following treatment will be tailored on following ctDNA reassessment
OPTIMIZE (NCT04680260) Denmark	Randomised Phase II Not yet recruiting	350 NA	• Radical intended treatment for metastatic CRC with no evidence of further disease • Clinically eligible for adjuvant chemotherapy • ctDNA-guided post-surgical treatment
DYNAMIC-II (ACTRN12615000381 583) Australia	Phase III Recruiting	450 NA	• Resected stage II CRC • Pts will be randomly assigned to ctDNA treatment-guided group or not, and to those ctDNA-positive 5-FU will be given while to ctDNA-negative will be followed up
DYNAMIC-III trial (ACTRN12617001566325) Australia	Randomised Phase II/III	1000 NA	• Resected stage III colon cancer • ctDNA-negative pts in experimental arm will be de-escalated adjuvant treatment strategy and those ctDNA-positive will be escalated adjuvant treatment strategy; control will be treated as per SoC
MEDOCC-CrEATE (NL6281/NTR6455) Netherlands	Randomised TwiCs design Recruiting	1320 NGS PGDx elio panel	• Stage II colon cancer pts without indication for adjuvant treatment according to current guidelines • ctDNA-positive pts will be offered 8 cycles of adjuvant capecitabine plus oxaliplatin while ctDNA-negative pts and control group will be followed up
COBRA (NCT04068103 and NRG-GI005) USA	Phase II/III Recruiting	1408 LUNAR panel	• Stage IIA resected CRC • Pts in experimental arm II will receive adjuvant treatment (at investigator choice) if ctDNA-positive and surveillance if ctDNA-negative
IMPROVE-IT (NCT03748680) Denmark	Phase II randomised Recruiting	64 NA	• Stage I or II disease radically resected • Detectable ctDNA in post-operative plasma sample • No indication for adjuvant chemotherapy according to DCCG guidelines but standard adjuvant chemotherapy administered if ctDNA-positive
(NCT03436563) USA	Phase Ib/II Recruiting	74 NA	• Pts with detectable ctDNA following resection of all known liver metastases will receive treatment with an anti-PD-L1/TGFbetaRII Fusion Protein M7824 • Resected MSS metastatic CRC
ALTAIR (NCT04457297) Japan	Phase III Recruiting	240 Signatera panel	• Pts who undergone radical curative resection of the primary and metastatic tumours • Pts tested positive for ctDNA but with no evidence of disease at imaging will receive TAS-102 or placebo
VEGA (jRCT1031200006) Japan	Phase III Recruiting	1240 NA	• High‐risk stage II or low‐risk stage III (T1‐3 and N1) CRC, and ctDNA‐negative status at week 4 after surgery • Randomisation between surgery alone versus adjuvant CAPOX
BESPOKE (NCT04264702) USA	NA Recruiting	2000 Signatera panel	• Resected stage II or III colorectal cancer (CRC) • Pts may be recommended for adjuvant treatment or observation by their treating clinician
*Neoadjuvant setting*			
SYNCOPE (NCT04842006) Finland	Randomised Not yet recruiting	93 NA	• LARC randomised to receive TNT using capecitabine/oxaliplatin and SCRT vs long course CRT using capecitabine • ctDNA and organoid-guided adjuvant therapy as experimental arm compared to SoC • Assessment of MRD after surgery and correlation with prognosis
*Metastatic unresectable disease*			
(NCT03844620) USA	Phase II Recruiting	100 NA	• Pts clinically eligible for either regorafenib or trifluridin-tipiracil • Pts will continue treatment beyond 1st cycle depending on ctDNA results
(NCT04831528) China	Phase II Not yet recruiting	100 NA	• Pts must have failed after first-line treatment containing cetuximab • Individualised second-line targeted therapy based on ctDNA analysis
FOLICOLOR (NCT04735900) International	NA Recruiting	60 NPY Methylation	• Unresectable metastatic disease • Identification of PD by NPY Methylation in liquid biopsies • To assess response and progression to first-line FOLFOX/FOLFIRI treatment on liquid biopsy
NCT04509635 China	Phase III Not yet recruiting	50 NA	• *RAS* wt on ctDNA • Non-resectable liver metastases candidate to anti-EGFR rechallenge based on ctDNA results
LIBImAb (NCT04776655) Italy	Phase III Not yet recruiting	280 *KRAS*, *NRAS* and in *BRAF*^*V600*^ status assessment using the Idylla system (Biocartis)	• *RAS/BRAF* wt on solid tumour biopsy but with *RAS* mutant at liquid biopsy • To compare di efficacy of FOLFIRI + Cetuximab or Bevacizumab in tissue wt but liquid mutant *RAS* mCRC
NCT04224415 China	Phase II Not yet recruiting	35 *RAS*/*BRAF* status assessment	• First-line therapy of FOLFOX/FOLFIRI/FOLFOXIRI + Cetuximab effectively and the PFS is not less than 6 months • ≥4 months after the last time treated with Cetuximab • *RAS/BRAF* wt on ctDNA
PARERE (NCT04787341) Italy	Phase II Recruiting	214 IdyllaTM ct*KRAS*-*NRAS*-*BRAF* Mutation Test	• *RAS* and *BRAF* wt status of primary CRC or related metastasis • *RAS* and *BRAF* wt ctDNA at the time of screening • Previous first-line anti-EGFR-containing therapy with at least a PR or SD ≥ 6 months; ≥4 months elapsed between the end of first-line anti-EGFR administration and screening; ≥1 line of therapy between the end of first-line anti-EGFR administration and screening
NCT04775862 Saudi Arabia	Phase II Recruiting	60 *RAS* status assessment	• Baseline must be *RAS/BRAF* wt on solid tumour tissue • *RAS* wt on ctDNA • Tumour burden with <4 organ involvement
NCT03992456 USA	Phase II Recruiting	120 Guardant360 assay	• *RAS* and *BRAF* wt on tumour tissue taken from primary or metastatic site • PD after treatment with an anti-EGFR monoclonal antibody for at least 4 months • ≥ 90 days from the last anti-EGFR treatment • *BRAF, EGFR, ERBB2, RAS, MET* wt highest allele frequency reported for any gene mutation <2%

These studies were retrieved through an extensive search performed on ClinicalTrial.gov in October 2021. The Medical Subject Headings terms used were (“Colo-rectal Cancer” as condition/disease) and (“circulating tumor dna” as other terms).

*ctDNA* circulating tumour DNA, *N* number, *pts* patients, *NA* not available, *CRC* colorectal cancer, *CAPOX* capecitabine plus oxaliplatin, *NGS* next-generation sequencing, *5-FU* 5-fluorouracil, *SoC* standard of care, *DCCG* Dutch Colorectal Cancer Group, *LARC* locally advanced rectal cancer, *SCRT *short course radiotherapy, *CRT* chemoradiotherapy, *MRD* minimal residual disease, *FOLFOX* 5-fluorouracil plus oxaliplatin, *TNT* total neoadjuvant treatment, *cCR* clinical complete response, *PD* progressive disease, *MSS* microsatellite stable, *NPY* Neuropeptide Y, *wt* wild-type.

^a^Matched historical control 1:3 with TOSCA trial patients.

### Neoadjuvant setting in locally advanced rectal cancer (LARC)

The current consensus on the management of locally advanced rectal cancer (LARC) below the peritoneal reflection consists of a multimodality treatment of neoadjuvant chemoradiotherapy (CRT) [67]. The pre-operative treatment of LARC, requiring a detailed pre- and post-treatment disease staging [68, 69], has contributed to decreasing the risk of local and distant relapse over time but has not disruptively changed the chance of survival [70].

More recently, several randomised trials have shown that pre-operative chemotherapy intensification as part of a total neoadjuvant treatment (TNT) strategy doubles the pathological complete response (pCR) rate achieved by conventional neoadjuvant chemoradiation (25 vs 12%) [71]. The doubling in pCR rate suggests that through TNT surgery might be avoided in a higher proportion of cases, paving the way towards a safer surgery-free “watch-and-wait” approach [72]. This expanding complexity in the management of LARC, poses pressing clinical questions including patients selection for different pre-operative treatments and early disease reassessment but, given the predictive importance of pCR for a non-surgical strategy, perhaps the most pivotal question relates to the timing and methodology for assessing the clinical complete response (cCR) after the completion of the neoadjuvant treatment. Tracking cancer in blood more than any other biological classifier, including the immunoscore [73], has the potential to fill the gaps of the unmet clinical needs in LARC [37], as suggested by the retrospective studies mentioned above. In the studies looking at pre-surgery or post-surgery liquid biopsy, a ctDNA-positive test was associated with an extremely high risk of recurrence and shortened survival reaching impressively results in double-digit hazard ratios for most studies (Table 1). Tie and colleagues, found a recurrence rate significantly higher in 13 and 19 of 159 patients with detectable ctDNA both after pre-operative treatment and after surgery, with a hazard ratio (HR) of 6.6 and 13, respectively [74]. Similarly, in a cohort of 72 patients undergoing TNT in the GEMCAD 1402 trial, pre-surgery ctDNA detected MRD in 15% and was significantly associated with shorter DFS (HR 4; *P* = 0.033) and OS (HR 23; *P* < 0.0001) [75]. In another study, the overall margin-negative, node-negative resection rate significantly doubled in 17 patients with undetectable versus 9 patients with pre-operative detectable ctDNA (88 vs 44%; *P* = 0.007) [76].

Notably, other studies have instead focused on the role of LB in monitoring early response during neoadjuvant therapy, using sequential samplings before, during and after the end of the pre-operative treatment. In the study by Khakoo and colleagues, ctDNA detection after pre-operative CRT was associated with primary tumour regression by magnetic resonance tumour regression grade (mrTRG) [77]. In the same work, the detection of ctDNA at any timepoint (pre-CRT, mid-CRT, or after CRT) was associated with shorter metastasis-free survival (MFS), fostering the way for an early prognostic evaluation during neoadjuvant treatment. Other studies found correlations between pre-operative ctDNA-positive rate after CRT and pathologic features after surgical resection such as pathologic ypT stage, tumour regression grade (TRG), and pathologic complete response (pCR) rate [78–80]. On the other hand, no correlation was identified between pre-operative ctDNA and pathologic response in patients receiving TNT, possibly reflecting the higher sensitivity of the method used in this work [75]. In addition, a currently ongoing trial (NCT04842006) is prospectively investigating the role of ctDNA in defining adjuvant approach after TNT in LARC (Table 2). As depicted in the upper right box of Fig. 1, ctDNA might play a key role when deciding for curative surgery versus non-operative management in patients achieving near clinical complete response (cCR) or cCR after neoadjuvant multimodal CRT, as assessed by imaging and endoscopy.

## Non-resectable advanced disease

The majority of studies investigated the use of LB in unresectable metastatic CRC patients. The first important finding in this setting is that the molecular landscape of tumours analysed using ctDNA or tissue samples is concordant in the vast majority of cases [9, 20, 81]. This was initially reported in a cohort of 106 mCRC patients, where ctDNA analysis through allele-specific quantitative PCR achieved 100% specificity and sensitivity in capturing *BRAF*^*V600E*^ mutations and specificity and sensitivity of 98% and 92%, respectively, in detecting six *KRAS* point mutation tested (*G12A, G12C, G12D, G12S, G12V, G13D*) [82]. Similarly, in a population of 100 mCRC patients the mutational status of *KRAS*, *BRAF* and *NRAS* in plasma samples achieved a 97% concordance in capturing “*RAS* pathway mutations” between solid tissue and blood was evaluated [9]. In both studies and others, discordant samples were linked to real biological differences secondary to intra-tumour inter-lesion heterogeneity, previous treatments and/or low burden of disease [9, 81, 82]. A liquid biopsy has the added advantage that ctDNA captures alterations occurring in multiple genes, specifically *EGFR*, *ERBB2*, *PIK3CA* or *MAP2K1*, unshadowing new potential targets for treatment as well as putative mechanisms of resistance to SoC targeted therapies such as anti-EGFR, anti-BRAF and anti-HER2 agents [9, 10, 12, 24, 24, 81, 83, 84]. In addition, and more recently, in a cohort of 232 CRC patients both solid tumour tissue and ctDNA were genotyped and an overall high concordance (84.9–100.0%) increased to near 100% (97.0–100.0%) when considering only clonal alterations (Fig. 1) [23]. Finally, through the GI-SCREEN network, the same authors demonstrated that ctDNA genotyping significantly shortens biomarker evaluation turnaround time (3 days versus 11 in standard pathological assessment) and increases screening efficiency for targeted agents trial enrolment (9.5% enrolment versus 4.1%) [23]. Similarly, the TARGET study conducted in different solid tumour including CRC patients showed that ctDNA can enhance patients enrolment into early phase clinical trials [83, 85].

Of note, even though CTCs isolation and analysis might offer the chance to study the CRC genome in its integrity rather than small fragments of DNA with potential further biological insights, currently ctDNA analysis represents the most effective strategy to assess mCRC molecular alterations in the advanced stage of disease [86]. Indeed, directly comparing the amount of ctDNA and CTCs in mCRC patients, we found that the median number of CTCs was 0 (ranging from 0 to 73) while the median amount ctDNA was 732,573 genome equivalent (GE, being the total number of fragments of cfDNA/mL)/mL (ranging from 174,774 to 174,078,615 GE/mL) [86]. Similarly, other studies confirmed the paucity of CTC in the blood of CRC patients and/or complexities of CTCs isolation in this setting [86–88].

### Disease monitoring and the Darwinian evolution model of CRC clones

**Fig. 2:**
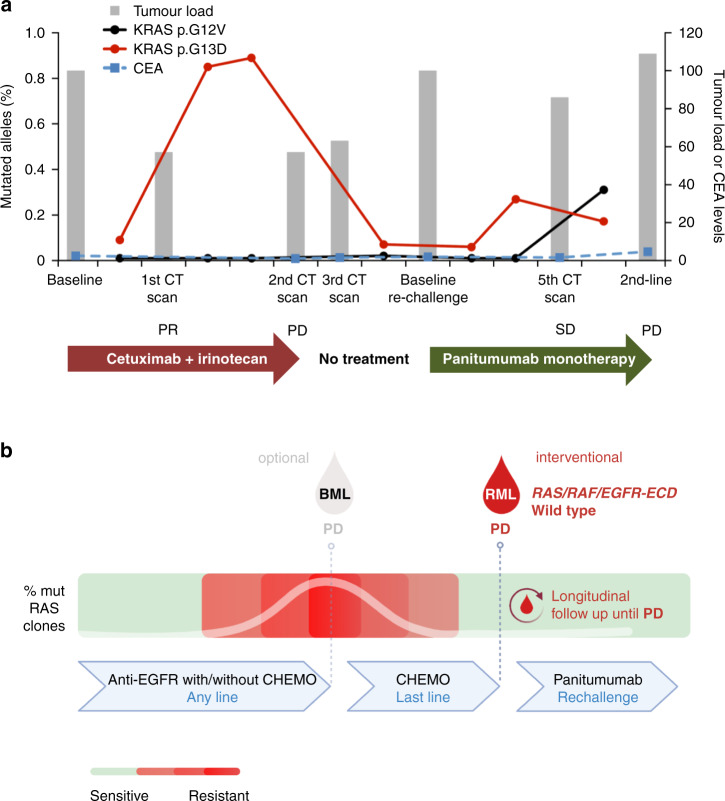
Tumour clones change consequently to drug-selective pressure. Specifically, mutated *RAS* mutant clones dynamically evolve in response to pulsatile EGFR-specific antibody administration in metastatic colorectal cancer (mCRC) patients. In the upper panel, the dynamic of *RAS* altered clones retrospectively monitored through circulating tumour DNA (ctDNA) of a mCRC patient ONCG-CRC69 (**a**)—adapted from Siravegna et al. Nat Med, 2015. Each treatment received by this patient are indicated. Grēy bars represent tumour load change during treatments. Tumour load was calculated as percentage change based on measurable disease at baseline assumed as 100%. Dotted blue line indicates changes in CEA values (ng/ml). Treatment outcome are reported according to RECIST criteria. Red lines indicate the frequency of *RAS* mutation (percentage of alleles) detected in circulating free DNA. In the bottom panel, a schematic representation CHRONOS clinical trial design (**b**). The CHRONOS trial is the first phase II trial prospectively aiming to assess the role of interventional ctDNA assessment to molecularly select mCRC patients towards rechallenge with anti-EGFR monotherapy. In this trial, mCRC patients *RAS*, *BRAF* and *EGFR* ectodomain wild-type on ctDNA received panitumumab monotherapy up to disease progression or toxicity. Finally, in this trial all enrolled patients are periodically and prospectively followed up for ctDNA collection to be retrospectively analysed to derive further exploratory translational data. Keys: CEA carcinoembryonic antigen.

In metastatic CRC, ctDNA was investigated as a tool to dynamically monitor the molecular evolution of CRC over time, under the pressure of different courses of treatment (Fig. 1) [9, 11, 20]. Indeed, we found that the amount of mutations conferring resistance to approved anti-EGFR agents is reflected by quantitative fluctuation and qualitative molecular landscapes change in ctDNA (Fig. 2a), revealing molecular evolution of CRCs which would have been impossible to assess by tissue biopsy [9, 24]. This pulsatile behaviour of tumour-specific mutant clones, identified through mutation monitoring over time on ctDNA, provided a scientific rational for the strategy of retreatment with anti-EGFR, previously attempted on clinical empiricism (Fig. 2b) [89]. Two studies showed retrospectively that mCRC patients harbouring *RAS* mutations on ctDNA achieved a significantly inferior response rate and progression-free survival when rechallenged with anti-EGFR agents if compared to those *RAS* wild-type on ctDNA (Table 1) [90, 91]. Both studies suggested that *RAS* assessment in ctDNA could improve the clinically-based selection of patients to be rechallenged with anti-EGFR retreatment [90, 91]. More recently, the CHRONOS trial prospectively confirmed this hypothesis (Fig. 2b) [92]. In CHRONOS, CRC patients approaching third or later line of treatment were assessed for *RAS*, *BRAF* and *EGFR* ectodomain status in ctDNA and rechallenged with anti-EGFR treatment only if a mutation-negative status was found [92]. Interestingly, by using this strategy a 30% response rate and a 63% disease control rate were achieved [92]. These figures favourably compare with those achieved by anti-EGFR rechallenge trials selecting patients empirically, and also by current SoC chemotherapies for the late disease space in mCRC [89–94]. Furthermore, CHRONOS supports the concept that a timely *RAS* assessment on ctDNA might be more reliable to select patients for anti-EGFR rechallenge than previously proposed mathematical models [92, 95]. Further albeit, retrospective correlative data on the role of ctDNA in the rechallenge setting are expected from an ongoing randomised phase III trial comparing third line standard of care versus anti-EGFR rechallenge strategy (AIO-KRK-0114; NCT02934529). In conclusion, liquid biopsy-driven rechallenge with anti-EGFR antibody monotherapy led to objective responses in one-third of mCRC patients. These results showed for the first time prospectively that genotyping tumour DNA in the blood of CRC patients can be used to direct therapy and can be effectively incorporated in the management of advanced CRC patients. As supported by initial prospective data and depicted in the lower-left box of Fig. 1, anti-EGFR rechallenge represents the real-world clinical scenario which will likely be impacted sooner by the introduction of interventional ctDNA assessment.

## Liquid biopsies for immunotherapy and beyond

All the applications discussed so far in CRC are focused either on the correlation between ctDNA presence and tumour burden (e.g. in the detection of MRD) or the identification of molecular alterations that predict response or resistance to targeted agents. However, recent developments in our understanding of the cancer genome and the increasing availability of sequencing technologies at progressively lower prices are paving the way for new biomarkers analysis also in ctDNA [14, 96].

TMB is presently being debated in CRC and other solid tumours given its correlation with response to immunotherapy and the recent Food and Drugs Administration (FDA) approval as an agnostic biomarker to access cancer immunotherapy with pembrolizumab or dostarlimab [97, 98]. TMB is defined as the number of mutations per megabase of DNA (Mut/Mb), and in CRC it is typically increased in case of microsatellite instability (MSI) or pathogenic mutations occurring in the proofreading domains of the DNA polymerases *POLE* and *POLD*, leading to a consequential increase of tumour neoantigens (TNA) likely driving response to immune checkpoint blockade [99]. It is noteworthy that a minority of TMB-high (≥20 Mut/Mb) cases occur also in MSS and *POLE/POLD* wild-type gastrointestinal cancers, mainly associated with mutations in other DNA damage response genes [100], although the effective response to immunotherapy for these tumours is yet to be prospectively evaluated and has shown inconsistent results across different retrospective analyses [101]. The gold standard for TMB evaluation is tumour tissue specimens [102] even if intra-tumour heterogeneity constitutes a relevant limit to its exact estimation, thus supporting the role of a ctDNA-based evaluation, as it already achieved in non-small cell lung cancer (NSCLC) [103]. Moreover, as for any other genetic or genomic biomarker, TMB can change under treatment with standard cytotoxic agents in CRC [104]. Thus, ctDNA-based evaluation of TMB as a criterion to predict response to immunotherapy after cytotoxic priming with temozolomide in O-6-Methylguanine-DNA Methyltransferase (*MGMT*) methylated mCRC, is currently being investigated in the ARETHUSA trial (NCT03519412) (Table 2 and Fig. 1). Importantly, even though this biomarker is promising, the chromosomal regions to be considered for its calculation, as well as the fractional abundance of supported mutations and its cut-off values are far from being standardised, despite many ongoing international efforts to harmonise the way TMB is analysed and reported [105].

On the other hand, MSI is currently the most relevant biomarker for immunotherapy sensitivity in CRC, typically assessed on solid tissue specimens [3, 4]. However, similarly to TMB, MSI status is subjected to both spatial and temporal heterogeneity [106], making its monitoring through LB therapeutically valuable.

Further potential exploitation of LB in CRC is the analysis of methylation biomarkers, which is rapidly emerging as a powerful methodology for early diagnosis and prognosis [20]. However, until epigenetic drugs reach the clinical setting in CRC, clear ctDNA interventional applications in this setting will be lacking [107]. In addition, another intriguing frontier in the analysis of ctDNA, although speculative at present time, is the study of mutational signatures as a proxy to identify cancer evolution and as predictive factors for treatment and/or the onset of resistance [108]. In summary, despite being of high potential translational value as depicted in the lower right box of Fig. 1, these ctDNA applications need further refinements before they can be deployed clinically.

## Overcoming limitations to interventional use of liquid biopsy in colorectal cancer

Despite the wealth of clinical opportunities offered by the analyses of ctDNA in CRC, logistical and biological limitations still limit its extensive application (Supplementary Table 1). The main logistical reason hampering the use of ctDNA-based analyses consists in their feasibility outside academic or comprehensive cancer centres [109]. One way to overcome this might be the centralisation of ctDNA analyses in selected referral centres, but this would require large cost-effectiveness studies and initiatives that need national and/or international public support. Conversely, using LB as a companion diagnostic could be particularly useful in those cases for which the result predicts response to medical treatment (targeted or immunotherapy) [110]. These two approaches could be complementary for the different settings, even though they would both require a simplification and standardisation of sample acquisition, in order to decrease pre-analytical biases as much as possible [111]. A second relevant logistical limitation for ctDNA analysis relies on the availability of different assays with different technical features in terms of sensitivity, LOD and reproducibility, whose accurate description is beyond the scope of this review since thoroughly discussed elsewhere [112]. Moreover, even if the industry has been a driving force in the development of the available LB platforms, we lack head-to-head comparisons of the different techniques for the same setting, making its cost-effectiveness evaluation difficult [113]. However, although no absolute preference can be given for one specific technology over the other, a balance between sensitivity required by the clinical question and widespread availability of the technique is advisable. For instance, less sensitive and/or automated platforms have shown good clinical performance in some settings [91, 114], while more sensitive and/or customised platforms, even based on a combination of different approaches, might be better for MRD detection [30, 115].

Biological limitations hampering ctDNA applicability into the clinic, must be analysed by considering other relevant CRC pathological, clinical and biological features (Supplementary Table 1). Tumour DNA shedding constitutes the first and more relevant of these limitations, and is known to be variable across stages, ranging between 73 and 100%, and primary tumour location in CRC [22, 116]. Moreover, DNA shedding not only correlates with tumour burden, similarly to other serum biomarkers such as CEA and CA19-9 [117], but also with the localisation of tumour metastases [118, 119]. In particular, the results from several studies investigating ctDNA-based analysis of *KRAS* mutations in mCRC have shown that the absence of liver metastases (e.g. in nodal or peritoneal-limited advanced disease) translates to reduced concordance with tissue analysis, that can be as low as 56% [120–122]. In a recent study, Bando and colleagues reported that patients with lung-only and peritoneum-only metastasis had significantly lower variant allele frequencies (VAFs) and lower numbers of detected variants, suggesting lower DNA release of subclonal variants in the blood [119]. Specifically, ctDNA was detectable in patients with lung-only metastases only in case of ≥20 mm of longest diameter and/or more than 20 lesions, and more than 20 mm of longest diameter in patients with peritoneum-only disease [119]. Collectively, these results evidence how low DNA shedding from lung- or peritoneal-limited disease could be considered another limitation to the application of liquid biopsies in patients with mCRC.

Moreover, the histopathological context of the cancer—that takes into account growth rate, stromal and inflammatory component, the extent of tumour cell death and necrosis—represents another determinant of cfDNA shedding that is intrinsically variable across CRC patients [123]. While the problem of DNA shedding affects mainly the sensitivity of LB, the presence of genetic aberrations in cfDNA originating from not cancerous tissues is a limitation to the specificity and sensitivity of circulating DNA analyses. Clonal Hematopoiesis (CH) is defined as the age-related accumulation of somatic mutations in hematopoietic stem cells which leads to clonal expansion of mutations in blood cells, and this is a primary source of false-positive results from ctDNA analysis [124]. CH is a relevant phenomenon that is reported in more than 10% of tumour-free patients over the age of 70 [125]. In a large dataset of more than 17,000 advanced cancer patients, it was shown that 5% of the patients would have at least 1 CH-associated mutation misattributed as tumour-derived in the absence of matched germline DNA sequencing [126]. The most common mutations derived from CH involve genes implicated in haematological tumorigenesis, such as *DNMT3A, TET2, ASXL1* and *JAK2*, but also genes frequently mutated in solid tumours such as *TP53, KRAS, PIK3CA* and *EGFR* can be often reported, potentially leading to misinterpretation of the actionability of cancer [81, 124]. A recent work by Huang and co-workers identified *KRAS* mutations in three mCRC patients pre-treated with chemotherapy to be CH-derived by paired peripheral blood cells (PBCs) sequencing, even if the fractional abundance of all these mutations were reported <5% [127]. The impact of CH in the detection of MRD was also investigated by Chan and colleagues, whereby 17% of the pre-operative cfDNA mutations were CH-related and recurrently detected after surgery or completion of adjuvant chemotherapy [128]. Collectively, these results indicate that paired peripheral blood marrow cells (PBCs) or solid tumour tissue sample (either from the primary tumour or a metastatic site) sequencing should be taken into consideration whenever ctDNA results influence therapeutic choices to rule out between CH and CRC specific alterations (Supplementary Table 1). Moreover, this limitation might be overtaken by exploiting barcoded DNA sequencing methods and integrating different approaches for ctDNA detection (i.e. using fragmentomics) to increase the capability of discriminating the CH-driven molecular background from cancer mutations derived from colorectal tumours.

At the present time, another limitation of performing extended ctDNA molecular panels in CRC patients is the lack of clear evidence that therapeutic intervention can be driven by liquid biopsy findings. Indeed, and differently from NSCLC [129], apart from the recently completed CHRONOS trial, there is a lack of evidence indicating the activity of a specific agent based on ctDNA analyses in CRC.

## Conclusions

Liquid biopsy is increasingly gaining traction in the clinical management of CRC patients in several clinical settings (Fig. 1). Retrospective data indicate that ctDNA can identify CRC patients requiring adjuvant treatments or conversely, not needing surgery after neoadjuvant treatment for LARC. Accordingly, once confirmed prospectively, the use of LB to detect MRD post-surgery with curative intent will likely be widely used in the management of early-stage CRC. Recently, the CHRONOS clinical trial demonstrated that ctDNA-based anti-EGFR rechallenge treatments can improve the therapeutic index of this therapeutic regimen. Presently, this is the only prospective and interventional evidence supporting the use ctDNA in CRC patients’ management, and accordingly anti-EGFR rechallenge is the setting in which ctDNA appears closer to clinical application. However as discussed above, ctDNA is likely to play a role also in selecting CRC potentially benefitting from other targeted therapies and immunotherapy, given its potential capability of capturing TMB and MSI features. In summary, even in presence of several biological and logistical limitations, LB will likely become central to rationally guiding CRC management. Ultimately, the accumulation of data from an ongoing perspective and randomised trials will determine the impact of ctDNA assessment for CRC patients’ care.

## Supplementary information

Supplementary table legends

Supplementary table 1

## Data Availability

Not applicable.
