# Supplementary material for: Liquid biopsies to monitor and direct cancer treatment in colorectal cancer
Source: Br J Cancer. 2022 Mar 9;127(3):394–407. doi: 10.1038/s41416-022-01769-8 (PMC9346106; doi:10.1038/s41416-022-01769-8)
Supplement: Supplementary file 1 — Supplementary table legends [file 41416_2022_1769_MOESM1_ESM.docx]

**Supplementary table legends**

**Supplementary table 1**. Main technical and biological limitations hampering the routine use of circulating tumour DNA (ctDNA) analysis in colorectal cancer (CRC), together with potential strategies to overcome them.
