# Supplementary material for: Liquid biopsies to monitor and direct cancer treatment in colorectal cancer
Source: Br J Cancer. 2022 Mar 9;127(3):394–407. doi: 10.1038/s41416-022-01769-8 (PMC9346106; doi:10.1038/s41416-022-01769-8)
Supplement: Supplementary file 2 — Supplementary table 1 [file 41416_2022_1769_MOESM2_ESM.docx]

**Supplementary table 1**. Main technical and biological limitations hampering the routine use of circulating tumour DNA (ctDNA) analysis in colorectal cancer (CRC), together with potential strategies to overcome them.

| **Limitation(s)** | **Description** | **Potential solution(s)** |
| --- | --- | --- |
| *Logistical and technical limitations* | | |
| Feasibility outside referral cancer centres | Limited access to LB in community hospitals outside clinical protocols, due to excessive costs of the assays and lack of ctDNA analysis technologies in house | - Centralization of analysis in referral cancer centres with standardized pre-analytical conditions - Use of ctDNA tests as companion diagnostic to access treatment |
| Different panels and methods available to collect and analyse ctDNA | Numerous available ctDNA assays with highly variable technical features in terms of sensitivity, specificity, and targets, without available direct comparisons. | - Simplification and standardization of samples acquisition - Standardization of the quality check pipeline - Balance between the sensitivity required by the clinical question and the availability of the technique |
| *Biological limitations* | | |
| Non-shedding CRC | Decreased DNA shedding into blood stream by around 20% of CRC (more frequently in rectal cancers, peritoneal limited metastatic CRC or low burden of disease limited to the thorax), with increased risk of false negative results | - Initial patients’ assessment and monitoring should not rely only on ctDNA but include ctDNA among other standard staging and diagnostic methods - Paired biopsy pre- and post-treatment/surgery for the identification of MRD - Consider tumour burden and localisation (e.g., absence of liver metastases drastically decreases DNA shedding) |
| Clonal haematopoiesis | Presence of circulating DNA mutations in cancer-related genes in PBCs derived from non-cancerous clones from the bone marrow, leading to potential false positive results | - Paired PBCs sequencing whenever ctDNA detects mutations at low allele frequency - Paired sequencing of both one solid tumour biopsy/surgery sample and ctDNA to identify specific trunk mutations |
| Lack of commonly accepted cut-off to define ctDNA positivity | Given different methods and panels, detection rates vary significantly | - The identification of specific cut-offs shared by different methodologies would be warranted |
| Lack of approved targeted treatments based on ctDNA molecular findings | Despite the opportunity to identify specific CRC molecular alterations, currently there are no approved treatment options based on ctDNA findings | - Clinical trials to guide targeted treatments based on interventional ctDNA molecular results are warranted |
| Concomitant identification of several genetic alterations | The use of more extensive panels unveils the co-occurrence of several genes alterations which are tough to interpret and prioritize for clinicians | - Providing comprehensive report might help clinicians in results interpretation towards improving clinical decisions |

Keys: LB=liquid biopsy; ctDNA=circulating-tumour DNA; CRC=colorectal cancer; MRD=minimal residual disease; PBMs=peripheral blood cells.
